# Knowledge on muscle strength among health professionals in Colombia: cross- sectional study

**DOI:** 10.15649/cuidarte.3953

**Published:** 2024-10-23

**Authors:** Gabriela Ruiz-Uribe, Jose P. Lopez-Lopez, Isabela Gómez-Montoya, Yuri Sanchez-Martínez, Mabel Reyes, Ana M. Gonzalez, Álvaro Castañeda-Hernandez, Daniel D. Cohen, Diego Gomez-Arbelaez, Johanna Otero, Daniel Martínez-Bello, Patricio Lopez-Jaramillo

**Affiliations:** 1 Universidad de Santander, Facultad de ciencias medicas y de la salud, Instituto de investigación Masira, Bucaramanga, Colombia. E-mail: mgruiz701@gmail.com Universidad de Santander Universidad de Santander Facultad de ciencias medicas y de la salud Instituto de investigación Masira Bucaramanga Colombia mgruiz701@gmail.com; 2 Universidad de Santander, Facultad de ciencias medicas y de la salud, Instituto de investigación Masira, Bucaramanga, Colombia. E-mail:josepatriciolopez@gmail.com Universidad de Santander Universidad de Santander Facultad de ciencias medicas y de la salud Instituto de investigación Masira Bucaramanga Colombia josepatriciolopez@gmail.com; 3 Universidad de Santander, Facultad de ciencias medicas y de la salud, Instituto de investigación Masira, Bucaramanga, Colombia. E-mail: isabelagomezm14@gmail.com Universidad de Santander Universidad de Santander Facultad de ciencias medicas y de la salud Instituto de investigación Masira Bucaramanga Colombia isabelagomezm14@gmail.com; 4 Universidad de Santander, Facultad de ciencias medicas y de la salud, Instituto de investigación Masira, Bucaramanga, Colombia. E-mail: ysancmartinez@gmail.com Universidad de Santander Universidad de Santander Facultad de ciencias medicas y de la salud Instituto de investigación Masira Bucaramanga Colombia ysancmartinez@gmail.com; 5 Universidad de Santander, Facultad de ciencias médicas y de la salud, Instituto de investigación Masira, Bucaramanga, Colombia. E-mail: ma.reyes@mail.udes.edu.co Universidad de Santander Universidad de Santander Facultad de ciencias médicas y de la salud Instituto de investigación Masira Bucaramanga Colombia ma.reyes@mail.udes.edu.co; 6 Universidad de Santander, Facultad de ciencias médicas y de la salud, Instituto de investigación Masira, Bucaramanga, Colombia. E-mail: go.ana@javeriana.edu.co Universidad de Santander Universidad de Santander Facultad de ciencias médicas y de la salud Instituto de investigación Masira Bucaramanga Colombia go.ana@javeriana.edu.co; 7 Universidad de Santander, Facultad de ciencias médicas y de la salud, Instituto de investigación Masira, Bucaramanga, Colombia. E-mail: al.castaneda@mail.udes.edu.co Universidad de Santander Universidad de Santander Facultad de ciencias médicas y de la salud Instituto de investigación Masira Bucaramanga Colombia al.castaneda@mail.udes.edu.co; 8 Universidad de Santander, Facultad de ciencias médicas y de la salud, Instituto de investigación Masira, Bucaramanga, Colombia. E-mail: danielcohen1971@gmail.com Universidad de Santander Universidad de Santander Facultad de ciencias médicas y de la salud Instituto de investigación Masira Bucaramanga Colombia danielcohen1971@gmail.com; 9 Universidad de Santander, Facultad de ciencias médicas y de la salud, Instituto de investigación Masira, Bucaramanga, Colombia. E-mail: diedgomez@gmail.com Universidad de Santander Universidad de Santander Facultad de ciencias médicas y de la salud Instituto de investigación Masira Bucaramanga Colombia diedgomez@gmail.com; 10 Universidad de Santander, Facultad de ciencias médicas y de la salud, Instituto de investigación Masira, Bucaramanga, Colombia. E-mail: johanna.otero.w@gmail.com Universidad de Santander Universidad de Santander Facultad de ciencias médicas y de la salud Instituto de investigación Masira Bucaramanga Colombia johanna.otero.w@gmail.com; 11 Universidad de Santander, Facultad de ciencias médicas y de la salud, Instituto de investigación Masira, Bucaramanga, Colombia. E-mail: dan.martinez@mail.udes.edu.co Universidad de Santander Universidad de Santander Facultad de ciencias médicas y de la salud Instituto de investigación Masira Bucaramanga Colombia dan.martinez@mail.udes.edu.co; 12 Universidad de Santander, Facultad de ciencias médicas y de la salud, Instituto de investigación Masira, Bucaramanga, Colombia. Universidad UTE, Facultad de Ciencias de la Salud Eugenio Espejo, Quito, Ecuador. E-mail: jplopezj@gmail.com Universidad de Santander Universidad de Santander Facultad de ciencias médicas y de la salud Instituto de investigación Masira Bucaramanga Colombia jplopezj@gmail.com

**Keywords:** Cross-Sectional Study, Muscle Strength, Knowledge, Grip Strength, Health Care Professionals, Estudios de Corte Transversal, Fuerza Muscular, Conocimiento, Fuerza de la Mano, Profesionales de la Salud, Estudos de Corte Transversal, Força Muscular, Conhecimento, Força da Mão, Profissionais de Saúde

## Abstract

**Introduction:**

Low muscle strength is a risk factor for various health conditions such as cardiometabolic diseases, neurodegenerative syndromes and mortality.

**Objective:**

Evaluate the knowledge of muscle strength in health professionals in Colombia. **Materials and Methods:** An analytical cross-sectional study was conducted in health professionals attending two continuing medical education events. Three components were evaluated through a questionnaire: identification of muscle strength as a risk factor for health conditions, measurement of muscle strength and education in muscle strength.

**Results:**

501 participants (52.49% women) were evaluated. Of these, 53.89% (n=270) were general practitioners, 18.16% (n=91) specialists and 6.18% (n=31) nurses. The association between low muscle strength and cardiometabolic diseases was identified by 56.67% (n=153) of general practitioners and 41.94% (n=13) of nurses. The indication for measuring muscle strength in older adults was recognized by 86.81% (n=79) of specialist physicians and 41.94% (n=13) of nurses. 32.93% (n=165) of the participants were aware of some method for measurement. Physiotherapists were the group that mostly reported measuring muscle strength by 83.33% (n=20). Only 29.03% (n=9) of the nurses had received academic information on muscle strength.

**Discussion and Conclusions:**

This study demonstrates the lack of knowledge on low muscle strength, its association with health conditions and measurement methods, and the lack of information about published literature on the subject. Educational interventions are needed to incorporate muscular strength evaluation into the clinical practice.

## Resumen.

**Introducción:**

La baja fuerza muscular es un factor de riesgo para diversas condiciones de salud como enfermedades cardiometabólicas, síndromes neurodegenerativos y mortalidad. **Objetivo:** Evaluar el conocimiento sobre fuerza muscular en profesionales de salud en Colombia.

**Materiales y Métodos:**

Se realizó un estudio analítico de tipo corte transversal en profesionales de salud asistentes a dos eventos de educación médica continuada. A través de un cuestionario se evaluaron tres componentes: identificación de la fuerza muscular como factor de riesgo para condiciones de salud, medición de la fuerza muscular y formación en fuerza muscular.

**Resultados:**

Se evaluaron 501 participantes (52,49% mujeres). 53,89% (n=270) eran médicos generales, 18,16% (n=91) especialistas y 6,18% (n=31). enfermeros. La asociación entre baja fuerza muscular con enfermedades cardiometabólicas fue identificada por el 56,67% (n=153) de médicos generales y 41,94% (n=13) de enfermeros. El 86,81% (n=79) de médicos especialistas y el 41,93% (n=13) de enfermeros reconocía la indicación de medir la fuerza muscular en adultos mayores. El 32,93% (n=165) de los participantes conocía de algún método de medición. Los fisioterapeutas fueron el grupo que más reportó realizar mediciones 83,33% (n=20). Sólo el 29,03% (n=9) de los enfermeros había recibido información académica sobre fuerza muscular.

**Discusión y Conclusión:**

Este estudio muestra el bajo conocimiento sobre la importancia de la fuerza muscular, su asociación a condiciones de salud, los métodos de medición y la falta de información sobre la literatura publicada al respecto. Son necesarias intervenciones educativas sobre fuerza muscular con el fin de incorporarla en la práctica clínica.

## Resumo.

**Introdução:**

A diminuição na força muscular é um fator de risco para diferentes condições de saúde, tais como doenças cardiometabólicas, síndromes neurodegenerativas e mortalidade.

**Objetivo:**

Avaliar o conhecimento sobre força muscular em profissionais da saúde na Colômbia.

**Materiais e Métodos:**

Realizou-se um estudo analítico de tipo transversal em profissionais da saúde que assistiram a dois eventos de educação médica continuada. Um questionário foi usado para avaliar três componentes: identificação da força muscular como fator de risco para condições de saúde, medição da força muscular e treinamento da força muscular.

**Resultados:**

Foram avaliados 501 participantes (52,49% mulheres). O 53,89% (n=270) eram médicos generalistas, 18,16% (n=91) médicos especialistas e 6,18% (n=31) enfermeiras. A associação entre força muscular diminuída com doenças cardiometabólicas foi identificada pelo 56,67% (n=153) dos médicos generalistas y 41,9% (n=13) dos enfermeiros. O 86,81% (n=79) dos médicos especialistas e o 41,94% (n=13) dos enfermeiros reconheceram a indicação de mensurar força muscular em idosos. O 32,93% (n=165) dos participantes conheciam algum método para mensuração de força muscular. Os fisioterapeutas foram o grupo que reportou realizar mensurações com maior frequência 83,33% (n=20). A principal causa para não realizar mensurações de força muscular foi a falta de equipamentos. Somente o 29,03% (n=9) dos enfermeiros tinham recebido informação acadêmica sobre força muscular.

**Discussão e Conclusão:**

Este estudo demostra o baixo conhecimento sobre força muscular diminuída e sua associação com condições de saúde, métodos de mensuração, e falta de informação sobre a literatura publicada. São necessárias intervenções educacionais para influenciar a prática clínica.

## Introduction

Muscle strength (MS) is defined as the tensile capacity that each muscle group can generate at a specific execution speed against a resistance^1^. Although there are several methods for its assessment, the most widely used technique is handgrip strength (HGS) by dynamometry^2^. HGS reflects the maximum force derived from the combined contraction of the extrinsic and intrinsic muscles of the hand^3^. According to the criteria established by the European Working Group on Sarcopenia in Older People (EWGSOP) ^4^, low HGS is defined as a measurement of > 2.5 standard deviations below the sex-specific population mean, as determined by dynamometry. While the majority of evidence about the correlation between low HGS and health conditions is primarily concentrated in the older adult population^5^^, ^^6^, recent data suggest this association is present throughout the life cycle^7^. Low HGS is a risk factor for cardiometabolic disease, neurodegenerative syndromes, and all-cause mortality in young and middle-aged adults^7^^, ^^9^. The UK Biobank study (n=493,774) showed that individuals in the lowest quartile of HGS had an increased risk of developing cardiometabolic disease (HR 1.46; 95% CI 1.34-1.60) and all-cause mortality (HR 1.87; 95% CI 1.64-2.14) compared to those in the highest quartile^10^. The growing acknowledgement of HGS as a clinical indicator of general and metabolic health has led to increased awareness and knowledge of the subject^11^^, ^^12^. It is, therefore, essential that healthcare professionals possess an adequate understanding of the role of MS in health status, as well as its implications for the treatment and prognosis of different conditions. However, the Sarcopenia Road Show demonstrated a limited awareness of MS. For instance, only 2% of healthcare professionals were able to correctly identify the cut-off points for determining low MS^13^^, ^^14^. Moreover, the primary barriers to routine assessment of MS identified were lack of awareness, unavailability of measurement equipment, and time constraints^13^^, ^^14^. The present study assessed the knowledge of MS as a risk factor for multiple adverse health events, its measurement, and the education received on this topic among medical and non-medical healthcare providers in Colombia.

## Materials and Methods

### A quasi-eStudy design, setting, and participants

A cross-sectional analyticalobservational study wasconducted accordingto the STROBEguidelines^15^. The population consisted of health professionals such as general practitioners, specialist physicians, nurses, physiotherapists, bacteriologists, and surgical instrument technicians. Participants and the population sample were selected on a convenience basis. Participants accessed the study via a QR code and completed the questionnaire on Google Forms, which automatically stored the responses in a database for further analysis. Data were collected at two different continuing medical education events held in Bucaramanga and Cartagena, Colombia, in August and October 2022, respectively. All participants completed an informed consent form stating that participation was anonymous and voluntary. This study was conducted during the preliminary phase of the study: “Efecto del entrenamiento de la fuerza isométrico en individuos con síndrome metabólico en su lugar de trabajo (EEFIT)”^16^. Ethical approval was granted by the Institutional Bioethics Committee of the University of Santander in minute No. 010 of May 10 and 15, 2018. This study was designed and developed in accordance with the principles of the Declaration of Helsinki. The data of this study are stored and accessible through Mendeley Data^17^.

### Variables

Three areas of interest were subjected to evaluation, including:

1. Identification of MS as a risk factor for disease, and the association of MS as
a risk factor for health conditions such as frailty, cardiometabolic diseases, and mortality. We sought to identify the age groups for which the measurement of MS was considered relevant. The age groups were defined in accordance with the life cycle delineated by the Colombian Ministry of Health and Social Protection^18^. The age categories were as follows: children (6 11 years), adolescents (12-18 years), young adults (14-26 years), adults (27-59 years), and older adults (60 years and above).
2. MS measurement (4 items) was evaluated. This assessed the participants'

knowledge, use of instrumental and non-instrumental measurement methods, reference values, measurement in routine clinical practice, and reasons for not including them in the physical examination of patients. 3) Health education was identified as to whether professionals had received continuing education in MS and health.

### Data sources / Measurements

Based on the three areas of interest outlined above, a survey-type questionnaire was developed comprising four multiple-choice questions and five dichotomous (yes/no) questions. In order to develop the survey, a literature review was conducted to support its content. Additionally, references on the level of knowledge in different areas of interest for health professionals were considered during its design^19^^, ^^20^. A preliminary validation of the questionnaire was conducted by three medical professionals to assess its clarity, coherence, and relevance. The suggested changes from the professional evaluators were made.

### Statistical analysis

The population was described by estimating means and standard deviations; categorical variables were described by counts and proportions. The normality of quantitative variables was assessed using graphical methods with histograms and numerical methods with the Shapiro-Wilk test. Categorical variables were compared using the chi-squared test; in the case of tables with expected values equal to or less than 5, Fisher's exact test was used. Bivariate analysis was performed to evaluate differences in strength knowledge and education by occupation using Pearson's chi-squared and Fisher's exact tests, with a statistical significance level (a) of 0.05. All analyses were performed using STATA 14 software.

## Results

During the study period, 501 participants completed the surveys. The sample was predominantly female with 52.49% (n=263), the majority identified themselves as general practitioners, accounting for 53.89% (n=270), followed by specialist physicians at 18.16% (n=91), physiotherapists at 4.79% (n=24), other health professionals at 16.96% (n=85), and nurses at 6.18% (n=31). The most common workplaces were hospitals/clinics 35.92% (n=180) and private practice 24.15% (n=121). (Table 1).

The category of other health professionals was composed of 60% (n=51) women, with a predominance of bacteriologists 8.23% (n=7), followed by students 7.05% (n=6), surgical instrument technician 5.88% (n=5), and speech therapists 1.17% (n=1) (Table S1).

**Figure 1:**
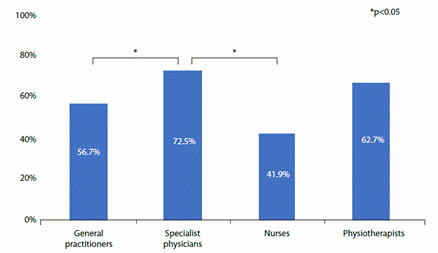
Knowledge of low muscle strength and risk of cardiometabolic disease by profession

When assessing knowledge of low MS and cardiometabolic disease risk by profession, specialist physicians were found to have a higher percentage of correct answers than general practitioners, nurses, and other professionals (p= 0.013). (Figure 1) (Table S2).

**Table 1:** Demographic characteristics of participants

Characteristics	(501)
Age Median (SD) - years	37.9 ± 13.7
Male- % (n)	47.05 (238)
Profession - % (n)	
General practitioners	53.89 (270)
Specialist physicians	18.16 (91)
Nurses	6.18 (31)
Physiotherapists	4.79 (24)
Other health professionals^3^	16.96 (85)
Workplace - n (%)	
Hospital/Clinic	35.92 (180)
Solo practice/ self-employed	24.15 (121)
University	18.96 (95)
Multidisciplinary practice	9.18 (46)
Pharmaceutical/commercial industry	4.79 (24)
Administrative/Government	1.79 (9)
Unemployed	0.19 (1)
Others3	4.79 (24)

a Other professionals include bacteriologists, surgical instrument technicians, speech therapists, students and other professionals who did not specify their training.

*P-value: Statistical significance (*a*) of 0.05, Pearson's Chi-Squared test*

**Figure 2:**
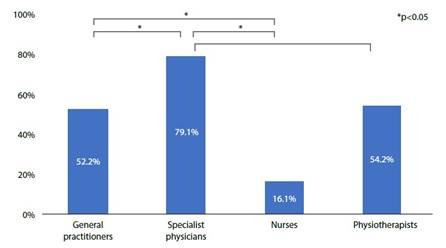
Knowledge of low strength and mortality by profession

Similarly, when assessing knowledge of low MS and mortality, specialist physicians had a higher percentage of correct answers than nurses and general practitioners (p= <0.001). (Figure 2).

*P-value: Statisticalsignificance (*a*) of 0.05, Pearson's Chi-Squared test*

**Figure 3:**
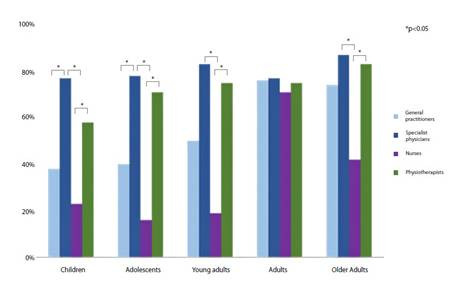
Knowledge about the importance of assessing muscle strength in different age groups by profession.

With regard to the population groups for which MS measurement is indicated, the age

group most frequently identified was adults. This group was identified by 378 professionals, of whom 76.92% (n=70) were specialist physicians and 75.93% (n=205) were general practitioners. Older adults were identified by 282 professionals, (86.81% (n=79) were specialist physicians, 83.33% (n=20), physiotherapists 83.33% (n=20) and general

physicians 74.44% (n=201)). The most frequently identified age group was adults, with 378 professionals (specialist physicians 76.92% (n=70), general practitioners 75.93% (n=205)) reporting this indication. Adolescents were identified less frequently, with 227 professionals (specialist physicians 78.02% (n=71), physiotherapists 70.83%, general physicians 40.00% (n=108)) reporting this indication. In children, it was reported by 225 professionals (specialist physicians 76.92% (n=70), physiotherapists 58.33% (n=14)) (p < 0.001) (Figure 3).

*P-value: Statistical significance (*a*) of 0.05, Pearson's Chi-Squared test*

59.15% (n=84) of general practitioners and 61.02% (n=36) of specialist physicians reported knowing some method of measuring MS. On the contrary nurses 77.78% (n=7) and in physiotherapists 75.00% (n=18) responded that they knew methods of measuring MS. (Table S3)

**Figure 4:**
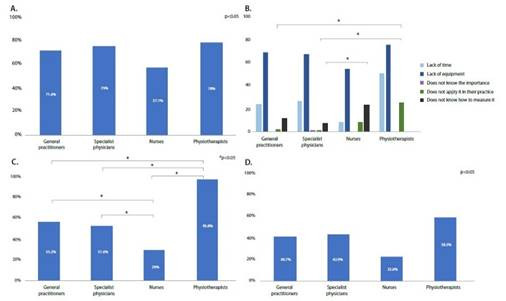
Muscle strength knowledge and education by profession

Instrumental measurement methods (dynamometry and bioimpedance) were identified similarly among all the groups evaluated. (Figure 4A).

*A. Knowledge ofinstrumental methods for muscle strength evaluation by profession. Reasons for not performing routine muscle strength measurement by profession. C.Academic training on strength and health by profession. D. Continuing health education on strength by profession. P-value: statistical significance (a) of 0.05, Pearson's Chi-Squared test*

Non-instrumental measurement methods were primarily identified by the group of physiotherapists (72.22%, n=13), while in the other groups, less than half of the respondents identified these methods. In addition, knowledge of reference values was known by 75.00 % (n=18) of the physiotherapists and by less than 30% of the participants in the other groups, general practitioners 24.07% (n=65), specialists 30.77% (n=28), and nurses 32.26% (n=10) (Table S3).

The main barriers to MS measurement were lack of equipment and time (Figure 4B). In the education component, physical therapists most frequently reported having received academic information on MS and health 95.83% (n=23), followed by general practitioners 55.18% (n=149), and specialist physicians 51.64% (n=47). The group that least reported having received academic information were nurses 29.03% (n=9) (p<0.001) (Figure 4C). Likewise, physical therapists most frequently reported having received continuing education at 58.33% (n=14), followed by specialist physicians at 42.85% (n=39) and general practitioners at 40.74% (110), and to a lesser extent, other professionals at 38.82% (n=33) and nurses 22.58% (n=7). (Figure 4D).

## Discussion

The findings of this study demonstrate that a significant number of health workers surveyed in Colombia exhibited limited awareness regarding the association between low MS and various health conditions. A high percentage of participants had no formal training or had not participated in continuing education. Although all participants identified low MS as a risk factor for at least one health condition, the association with cardiometabolic disease and mortality was only identified by a high proportion of specialist physicians. A high percentage of participants reported knowing some method of measuring MS, with instrumental methods (dynamometry and bioimpedance) being the most frequent. Finally, less than half of the participants, with the exception of physiotherapists, knew the reference values for diagnosing low MS, and few performed measurements as part of their daily practice, mainly due to lack of equipment and time.

This is the first study in Colombia to assess the knowledge and practices of MS measurement among health personnel. Similar out comes have been observed in studies conducted in high-in come countries. The Sarcopenia Roadshow is a continuing education program designed to strengthen strategies for the diagnosis and management of sarcopenia in the Netherlands, Australia, and New Zealand^13^^, ^^14^. In Australia and New Zealand, a high percentage of surveyed participants (81.4%) demonstrated knowledge of the diagnosis of MS. However, only 12% (n=30) of these individuals applied diagnostic methods in their daily practice. Regarding MS measurement methods, 33.9% (n=75) of health professionals in the Netherlands utilized HGS^14^. Our findings indicate that approximately 50% of the participants reported familiarity with a measurement method, with the most frequently selected method being HGS. Only a small percentage (2.0%), of Australian and New Zealand professionals demonstrated an accurate understanding of the diagnostic values for low MS as assessed with HGS. In our study, the percentages of knowledge were similarly low, except for the physiotherapist group. The primary obstacles to the implementation of diagnostic strategies, as reported by 62.7% of Dutch professionals, were identified as a lack of knowledge and equipment, as well as time constraints^14^. Similarly, 77.8% of Swiss nutritionists reported a lack of instruments, and 78.6% cited a lack of knowledge to perform the assessments^11^.

A study conducted in the United States that evaluated health personnel with a high probability of caring for patients with low MS in primary care demonstrated that 35% exhibited limited knowledge of FM^21^. In Colombia, the equivalent of these personnel are general practitioners. Despite a high percentage of respondents indicating familiarity with the concepts, only 17.41% (n=47) utilize them in their practice, and only 24.07% (n=65) employ standardized definitions for diagnosis^22^.

With regard to the continuing education of healthcare personnel, over half of the participants indicated that they had not yet received training or continuing education on reference values or methods of measuring MS. This is reflected in the low percentage of professionals who routinely assess MS. Similarly, in Australia and New Zealand, the Sarcopenia Road Show evaluated the retention and implementation of knowledge following educational activities. The results indicated that only 53.8% of respondents reported applying the concepts they learned in practice following a single educational event, suggesting low retention and application of the concepts after a single educational event^13^. The results of this study indicate that a combination of educational strategies should be employed to facilitate the integration of knowledge and to reinforce its practical application. This presents a challenge in training health professionals, as physicians, nurses, physiotherapists, and other professionals should perform MS assessments.

Finally, it is worth highlighting the usefulness of conducting this type of study to identify weaknesses in the training of health professionals in our region and in the care provided to people in different settings. In this regard, a survey conducted in Latin America to evaluate knowledge and beliefs about prediabetes among health professionals demonstrated a need to enhance knowledge about prediabetes, its clinical implications, and its treatment^15^. The results were found to be highly comparable to those observed in our own study. Consequently, it is imperative to cultivate a greater understanding among health professionals about the prevalent conditions and risk factors affecting the population and instill a sense of urgency for developing and implementing educational tools to address these gaps.

## Limitations

The study has some limitations, including the use of a survey with an incipient process of validation of the questionnaire and a lack of evidence for its validation. Another limitation is that most of the participants surveyed were physicians attending medical congresses, which may have given rise to a selection bias. Additionally, the sample included was selected foEDr convenience, which limits the generalizability of the results; therefore, more studies are needed that include more people who are not health personnel. One of the strengths of this study is that it provides, for the first time in Colombia, insight into the knowled.

## Conclusion

This study demonstrates that the knowledge and practices of healthcare professionals on MS are deficient, so there is a need to increase knowledge through educational interventions that can be incorporated into clinical practice. Future research is needed to evaluate the impact of increased knowledge on better management of conditions associated with muscle strength loss.

## Appendix

**Table S1:** Demographic characteristics of other health professionals

Characteristics	(85)
Age Media (SD) - yeas	34.4 ± 12.27
Male - % (n)	40.00 (34)
Profession - % (n)	
Bacteriologists	8.23 (7)
Surgical instrument technician	5.88 (5)
Speech therapists	1.17 (1)
Students	7.05 (6)
Othersa	77.64 (66)
Workplace - n (%)	
Hospital/Clinic	16.47 (14)
Solo practice/ self-employed	9.41 (8)
University	36.47 (31)
Multidisciplinary practice	4.70 (4)
Pharmaceutical/commercial industry	15.29 (13)
Administrative/Government	7.05 (6)
Unemployed	1.17 (1)
Othersa	9.41 (8)

^a^ *Others refer to professionals who did not specify their level of training*

**Table S2:** Identification of muscle strength as a risk factor

Answer	General Practitioners	Specialist Physicians	Nurses	Physiotherapist s	Other professionals^3^	P value
	(270)	(91)	(31)	(24)	(85)	
Muscle strength as a risk factor for disease						
Frailty b	84.07 (227)	89.01 (81)	58.06 (18)	87.50 (21)	74.11 (63)	0.001
Cardiometabolic disease ^c^	56.67 (153)	72.52 (66)	41.93 (13)	66.66 (16)	54.11 (46)	0.013
Mortality ^d^	52.22 (141)	79.12 (72)	16.12 (5)	54.16 (13)	43.52 (37)	<0.001
Important age group to measure muscle strength						
Children	38.51 (104)	76.92 (70)	22.58 (7)	58.33 (14)	35.29 (30)	<0.001
Adolescents	40.0 (108)	78.02 (71)	16.12 (5)	70.83 (17)	30.58 (26)	<0.001
Youth	49.62 (134)	83.51 (76)	19.35 (6)	75.0 (18)	43.52 (37)	<0.001
Adults	75.92 (205)	76.92 (70)	70.96 (22)	75.0 (18)	74.11 (63)	0.967
Older adults	74.44 (201)	86.81 (79)	41.93 (13)	83.33 (20)	62.35 (53)	<0.001

a*: Other professionals, including bacteriologists, surgical instrument technicians, speech therapists, students and other professionals who did not specify their training. b: Fragility includes fragility and osteoporosis. c: Cardiometabolic diseases include diabetes, hypertension, and cardiovascular disease. d: Mortality includes cardiovascular mortality and all-cause mortality*

**Table S3:** Strength Measurement

Question	General Practitioners	Specialist Physicians	Nurses	Physiotherapists	Other professionals ^a^	P value
	(270)	(91)	(31)	(24)	(85)	
Knowledge of methods for the estimation and/or measurement of muscular strength	59.15 (84)	61.02 (36)	77.78 (7)	75.00 (18)	66.67 (20)	0.485
Knowledge of methods for the estimation and/or measurement of muscle strengthb						
Instrumental methods^0^	71.43 (60)	75.00 (27)	57.14 (4)	77.78 (14)	70.00 (14)	0.865
Non-instrumental methodsd	34.52 (29)	25.00 (9)	42.86 (3)	72.22 (13)	45.00 (9)	0.014
Knowledge of and access to reference values for the measurement of muscle strength by some method	24.07 (65)	30.77 (28)	32.26 (10)	75.00 (18)	27.06 (23)	<0.001
Routine measurement of muscle strength or muscle function during patient care.	17.41 (47)	20.88 (19)	16.13 (5)	83.33 (20)	17.65 (15)	<0.001
Barriers to muscle strength measuremente						
Lack of time	23.32 (52)	26.39 (19)	7.69 (2)	50.00 (2)	15.71 (11)	0.107
Lack of equipment	68.61(153)	66.67 (48)	53.85 (14)	75.00 (3)	55.71 (39)	0.229
Does not know the importance	0 (0,0)	1.39 (1)	0.0 (0)	0.00 (0)	0.00 (0)	0.343
Does not apply it in their practice	2.24 (5)	1.39 (1)	7.69 (2)	25.00 (1)	4.29 (3)	0.041
Does not know how to measure it	13.45 (30)	8.33 (6)	26.92 (7)	0.00 (0)	24.29 (17)	0.006

*1-RM: One maximum repetition.* a*: Other professionals including bacteriologists, surgical instrument technicians, speech therapists, students and other professionals who did not specify their training. b: Response conditional on those who answered YES in the previous question. c: Instrumental methods include dynamometry and Bioimpedancemetry. d: Non instrumental methods include Daniels scale, manual muscle testing, strength/endurance/balance, lift and gait, 1-RM, exercise and other scales. e: Response conditional on those who answered NO in the previous question*
